# Anais Brasileiros de Dermatologia: adjustments to publication standards for authors. Expansion of Editorial Boards. Considerations about bibliometric analyses^⋆^

**DOI:** 10.1016/j.abd.2023.11.001

**Published:** 2023-12-18

**Authors:** Silvio Alencar Marques, Ana Maria Roselino, Hiram Larangeira de Almeida Jr, Luciana P. Fernandes Abbade

**Affiliations:** a Department of Infectology, Dermatology, Imaging Diagnosis and Radiotherapy, Universidade Estadual Paulista, Botucatu, SP, Brazil; b Division of Dermatolody, Department of Medical Clinics, Universidade de São Paulo, Ribeirão Preto, SP, Brazil; c Department of Dermatology, Universidade Federal de Pelotas, Pelotas, RS, Brazil; d Department of Dermatology, Universidade Católica de Pelotas, Pelotas, RS, Brazil

Dear Associates,

The Anais Brasileiros de Dermatologia (ABD) continues to update its standards for authors and promote changes in our continuing search for greater quality and visibility, both national and international, of the scientific articles we publish. Below are listed changes/additions to the standards for publication in the ABD.

Authorship: we eliminated the limitation to the number of authors in Research Letters, always trusting the academic rigor practiced by the authors.

References: DOI information is now mandatory for each reference when submitting an article, with no changes in the current reference system used and available on the journal websites.

Editorial Boards: in recognition of the academic contributions and to ABD in particular, provided by associates Mirian Nacagami Sotto, Cacilda da Silva Souza, Adriana Maria Porro, Neusa Yuriko Sakai Valente, we have invited them to be part of the National Editorial Board. Similarly, for the International Editorial Board, we have invited Jeffrey B Travers (USA), Raul Cabrera (Chile) and Jorge Ocampo-Candiani (Mexico).

We also highlight observations about the bibliometric analyses related to ABD. Recently published and available on the website, there are two educational and interesting articles regarding the main metrics used in the evaluation of scientific journals. These articles show data that support an extremely important discussion of the future of ABD and Latin American scientific production.^1^, ^2^ It is never too much to recall that we are the only journal in Dermatology in Latin America indexed in PubMed/Medline and we try to accomplish this responsibility by publishing good articles with special emphasis on articles focused on tropical endemic and neglected diseases.^3^, ^4^, ^5^ To successfully fulfill this mission we need the support of the Brazilian Society of Dermatology and, moreover, the support and academic recognition of several Dermatology societies from the countries that constitute the Latin America.

Thus, we call on associates and the scientific community in general to submit their articles to our journal, access our websites, publicize and support, whenever relevant, the Anais Brasileiros de Dermatologia.

https://www.anaisdedermatologia.org.br/

https://www.sciencedirect.com/journal/anais-brasileiros-de-dermatologia

## Financial support

None declared.

## Authors' contributions

Silvio Alencar Marques: Approval of the final version of the manuscript; drafting and editing of the manuscript.

Ana Maria Roselino: Approval of the final version of the manuscript; drafting and editing of the manuscript.

Hiram Larangeira de Almeida Junior: Approval of the final version of the manuscript; drafting and editing of the manuscript.

Luciana P. Fernandes Abbade: Approval of the final version of the manuscript; preparation and writing of the manuscript.

## Conflicts of interest

None declared.
